# Blood Flow Contributions to Cancer Metastasis

**DOI:** 10.1016/j.isci.2020.101073

**Published:** 2020-04-18

**Authors:** Francesc Font-Clos, Stefano Zapperi, Caterina A.M. La Porta

**Affiliations:** 1 Center for Complexity and Biosystems, Department of Physics, University of Milan, Via Celoria 16, 20133 Milano, Italy; 2 CNR - Consiglio Nazionale delle Ricerche, Istituto di Chimica della Materia Condensata e di Tecnologie per l’Energia, Via R. Cozzi 53, 20125 Milano, Italy; 3 Center for Complexity and Biosystems, Department of Environmental Science and Policy, University of Milan, via Celoria 26, 20133 Milano, Italy; 4 CNR - Consiglio Nazionale delle Ricerche, Istituto di Biofisica, via Celoria 26, 20133 Milano, Italy

**Keywords:** Biological Sciences, Mathematical Biosciences, Cancer

## Abstract

The distribution patterns of cancer metastasis depend on a sequence of steps involving adhesion molecules and on mechanical and geometrical effects related to blood circulation, but how much each of these two aspects contributes to the metastatic spread of a specific tumor is still unknown. Here we address this question by simulating cancer cell trajectories in a high-resolution humanoid model of global blood circulation, including stochastic adhesion events, and comparing the results with the location of metastasis recorded in thousands of human autopsies for seven different solid tumors, including lung, prostate, pancreatic and colorectal cancers, showing that on average 40% of the variation in the metastatic distribution can be attributed to blood circulation. Our humanoid model of circulating tumor cells allows us to predict the metastatic spread in specific realistic conditions and can therefore guide precise therapeutic interventions to fight metastasis.

## Highlights

• We construct a high-resolution humanoid model of blood circulation
• We use the model to simulate trajectories of circulating tumor cells
• We compile statistics of the distribution of metastatic sites predicted by the model
• Comparison with autopsies shows that flow accounts for 40% of metastasis distribution

## Graphical Abstract

**Figure:**
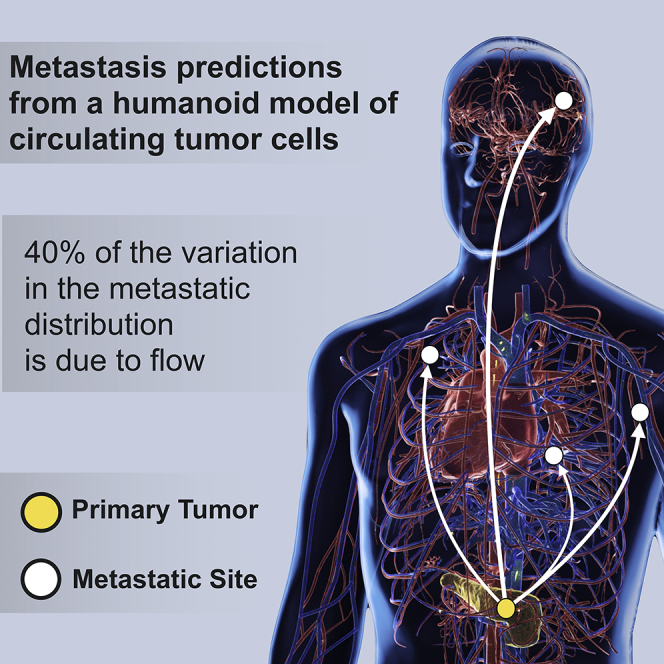

## Introduction

Cancer is the second cause of mortality worldwide, and metastasis is the main reason for patient death. The metastatic process is due to the spread of tumor cells through blood and/or lymphatic vessels and the capability of cancer cells to colonize specific sites. Already in 1889 (Paget, 1889), Paget claimed that metastasis does not occur by chance but only when tumor cells (the seeds) can adapt to a permissive microenvironment (the soil) of a given organ, as with seeds needing a fertile soil to grow and flourish. This point of view is supported by a vast literature (Fidler, 2003, Fokas et al., 2007). Later in 1929, Ewing stressed the importance for metastatic dissemination of mechanical and geometrical factors resulting from the anatomical structure of the vascular system and the associated hemodynamic flow (Ewing, 1919), a view also supported by experimental evidence (Weiss et al., 1980, Weiss, 1992) and computational models (Scott et al., 2014, Poleszczuk et al., 2016). Although both mechanical and seed-soil compatibility factors should play a role in the spread of metastasis (Chambers et al., 2002, Wirtz et al., 2011), the relative weight of each factor for a given cancer type and target organ is unknown owing to the lack of appropriate quantitative tools.

Here we build a high-resolution global blood circulation model of a humanoid male subject (Quarteroni, 2006, Müller and Toro, 2014, Blanco et al., 2014, Blanco et al., 2015, Huang et al., 2018), including stochastic adhesion events, to simulate the trajectories of circulating tumor cells (CTCs). Using the model, we estimate the colonization patterns of CTCs at the different target organs. We compare the simulation results with a statistical analysis of thousands of human autopsies reported in the literature (Abrams et al., 1950, Disibio and French, 2008, Bubendorf et al., 2000, Budczies et al., 2015, Schlageter et al., 2016) for seven primary tumors: lung, colorectal, prostate, pancreatic, liver, kidney, esophageal, and gastric cancers. The model allows one to estimate the contribution of geometrical and flow factors to the spread of metastasis, providing an essential guidance to interpret experimental data.

## Results

**Figure 1:**
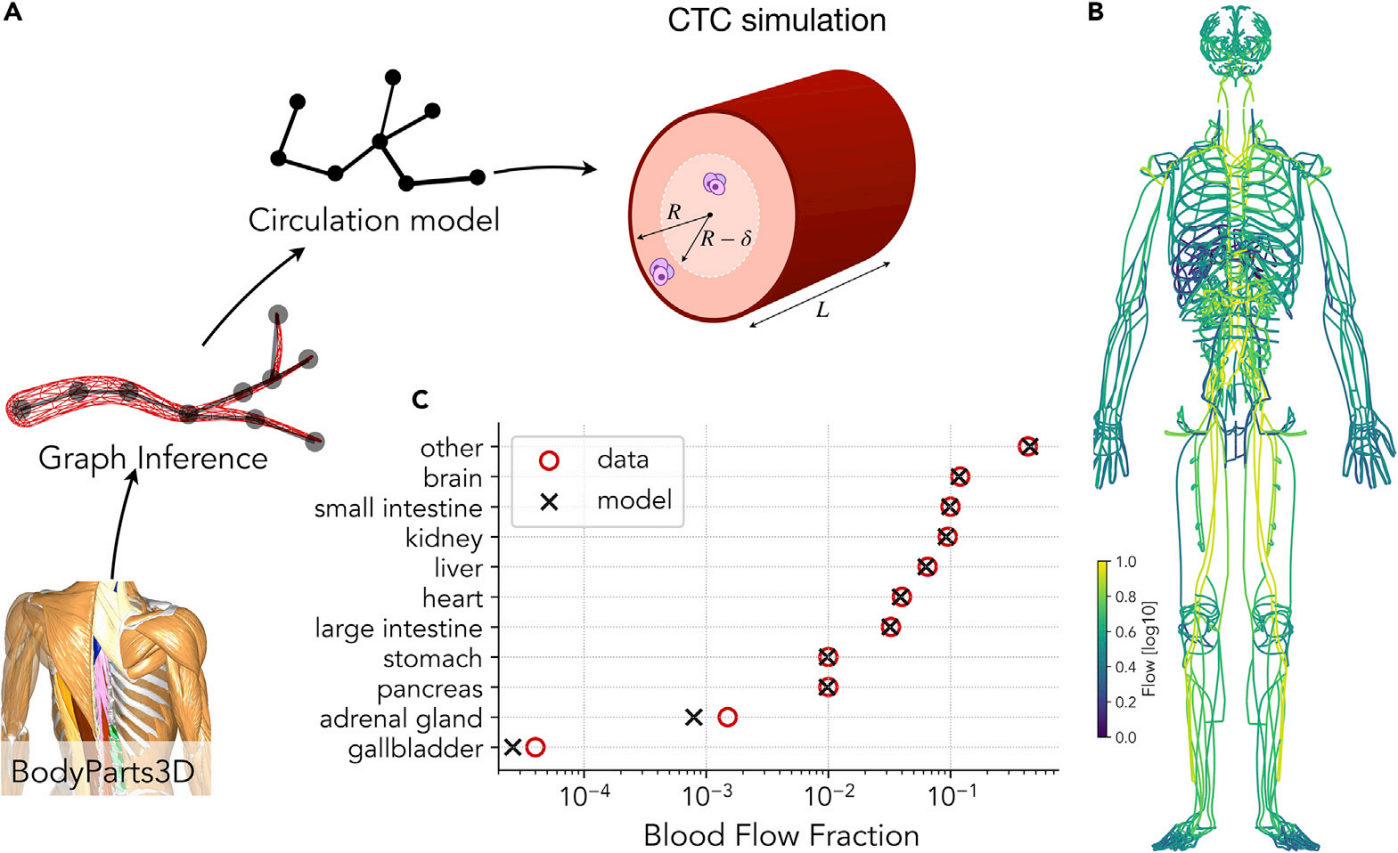
Computational Model of Global Arterial-Venous Circulation Reproduces Experimentally Measured Blood Flow Distribution (A) The circulation network is reconstructed from the meshes obtained from a 3D body scan. The blood flow is then obtained by imposing flow conservation and the Hagen-Poiseuille equation. CTC trajectories follow the blood flow and can attach to the vessel walls if they are within a distance δ of them. (B) Blood flow distribution obtained from the model. (C) The fraction of blood reaching each organ obtained with the model is in good agreement with experimental data from Williams and Leggett (1989).

We build an accurate network representation of arterial and venous circulatory systems starting from a full 3D whole-body model obtained from MRI images of a male subject taken at 2-mm resolution (BodyParts3D [Mitsuhashi et al., 2009]). From this model, we extract a set of 639 artery meshes, 395 vein meshes, and 16 organ meshes, and, using graph inference algorithms, we obtain a network composed by 23,285 nodes and 23,804 edges, each annotated with their radius *R* and length *L* (see Figure 1A). A morphometric analysis of the model is summarized in Figure S1A illustrating the decrease of vessel radius as a function of the generation number (see Methods section).

Blood flow patterns are then obtained by imposing flow rate conservation at each node and a pressure boundary condition across the hearth. The pressure drop Δp across each vessel is proportional to the flow rate *J* according to the Hagen-Poiseuille equation (see Figure 1A and Methods for additional details). From the solution of the hemodynamic flow equations, we compute flow distributions (Figure 1B) ensuring that the fraction of blood flow reaching each organ and the results compare well with experimental data (Figure 1C). Furthermore, the dependence of the arterial blood pressure on the vessel radius reported in Figure S1B is in agreement with physiological measurements (Guyton and Hall, 1986).

**Figure 2:**
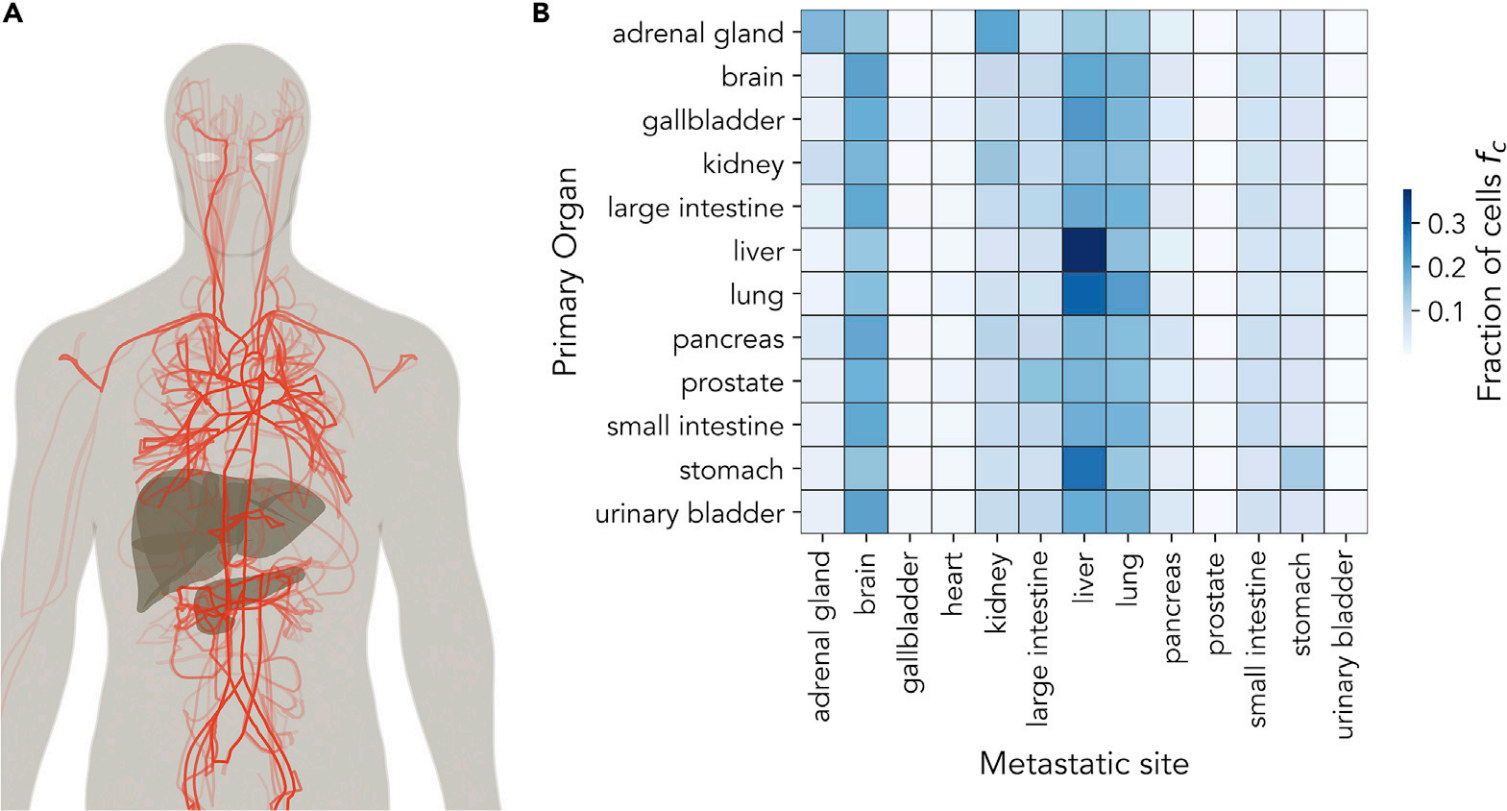
Model Simulations Allow Us to Estimate the Contribution of Flow and Geometric Factors to the Metastatic Distribution (A) Example of 30 simulated cancer cell trajectories released from the pancreas. (B) A color representation of the matrix , quantifying the fraction of simulated cell trajectories released from primary tumor *P* reaching the target organ *O*.

In our model, CTC trajectories are computed assuming that cancer cells are randomly released from the primary tumor and then follow the blood stream, randomly choosing the direction to take at each intersection with a probability that is proportional to the relative flow in each branch. Cancer cells can exit the blood stream with a probability that only depends on geometrical and hemodynamic factors as discussed in the Model section. Owing to computational limitations, we do not directly model the flow inside capillary beds but we estimate the probability to exit the blood stream from the typical geometry of capillary beds (see Figure S1C). In this way, the model does not consider specific microenvironmental seed-soil compatibility factors between cancer cells and target organs but only the geometry of capillary beds, where adhesion and extravasation are expected to occur. Examples of cancer cell trajectories released from the pancreas are reported in Figure 2A (see also Video S1).

Video S1. The Humanoid Vascular Model, Related to Figure 2 An illustration of the humanoid vascular model. Simulations refer to the spread of circulating tumor cells from the pancreas.

Simulating the model, we collect a large set of cell trajectories starting from a predetermined set of primary tumor sites P (i.e., lung, colon, prostate, pancreas, stomach, kidney, and liver) and determine the fraction of cells $f_{c}$ whose trajectory ends at a specific target organ O (including brain, liver, lungs, hearth, kidney, and pancreas). We launch a total of N = 10,000 trajectories starting from each organ, let them flow along the circulatory system, and wait until they eventually stop. In this way, we can infer the distribution of metastatic dissemination expected if only flow and geometric factors were present. A summary of the simulated pattern is reported in Figure 2B, showing the probability that a primary tumor P would metastasize at organ O.

To compare our numerical estimates with real metastatic dissemination patterns, we collect data on metastasis distributions from human autopsies reported in the literature (Abrams et al., 1950, Disibio and French, 2008, Bubendorf et al., 2000, Budczies et al., 2015, Schlageter et al., 2016). We restrict our analysis to studies originating directly from patient autopsies (Abrams et al., 1950, Disibio and French, 2008, Bubendorf et al., 2000, Budczies et al., 2015, Schlageter et al., 2016), disregarding other studies inferring metastatic patterns from medical records only (Chen et al., 2009, Qiu et al., 2015, Riihimäki et al., 2018). A collection of existing published data is summarized in Figures S2 and S3. For each primary tumor P, we report the fraction of patients $f_{p}$ with metastasis in organ O. The data show that there is a good consistency between the value of $f_{p}$ measured in different studies, taking into account expected uncertainties due to the sample size in each study. Nevertheless, we observe variations among studies that could be associated to a variety of factors intrinsic to each study, such as the location or the time at which data were gathered or the specific drug treatment of the patients. We generically refer these variations as “measurement errors.”

**Figure 3:**
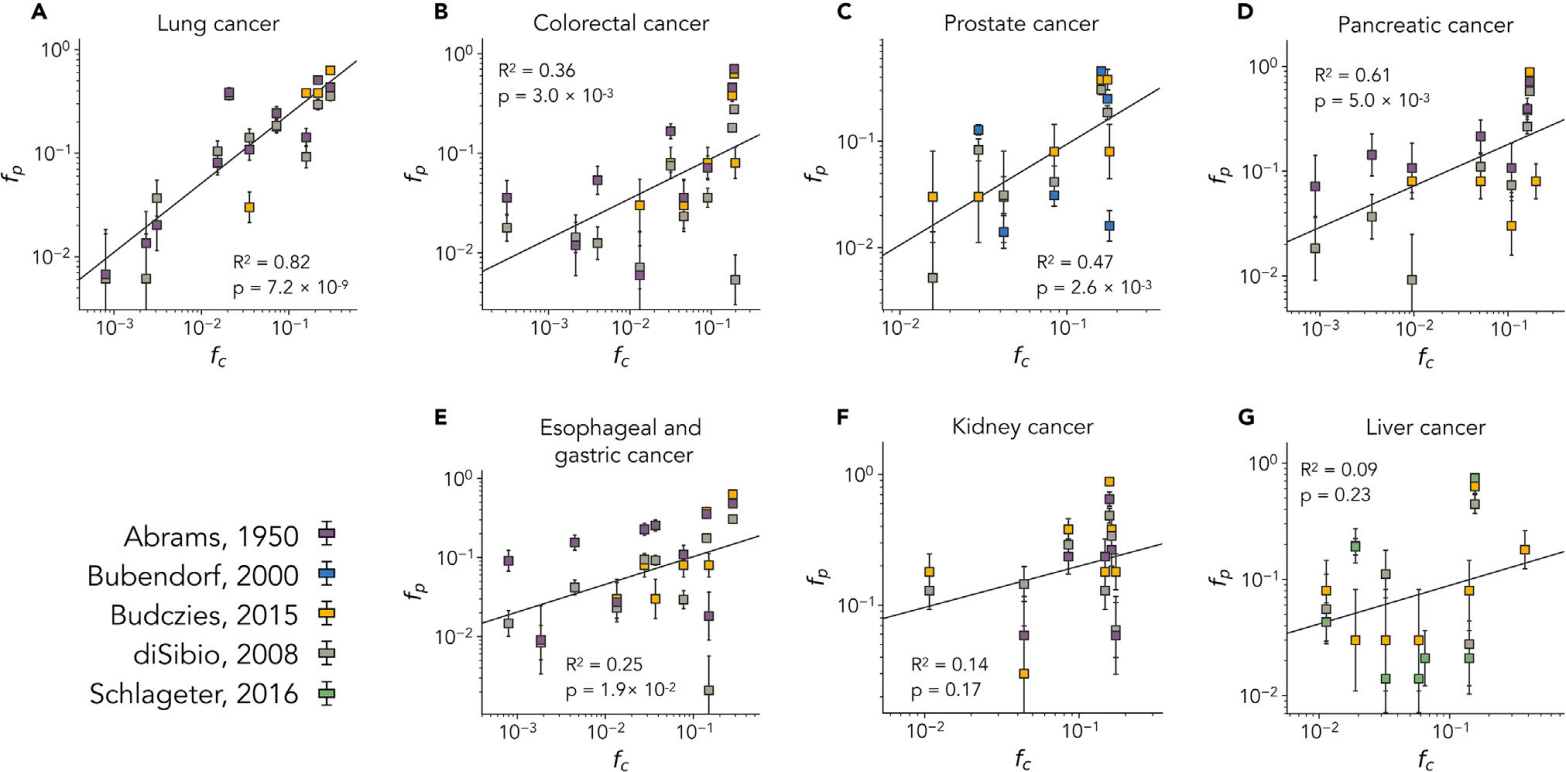
Distribution of Metastasis from Autopsies Correlates with Cancer Cell Dissemination Patterns Obtained from the Model Cross-correlation analysis of the fraction of patients $f_{p}$ with primary tumor *P* and metastasis found in a distant organ *O* and the corresponding fraction of simulated cell trajectories $f_{c}$ released from primary tumor *P* reaching the target organ *O*. Each plot corresponds to metastasis originating from a single cancer: (A) lung, (B) colorectal, (C) prostate, (D) pancreatic, (E) esophageal and gastric, (F) kidney, and (G) liver cancer. The fraction of patients $f_{p}$ is obtained from different studies, as reported in the legend. Error bars are standard errors estimated using a binomial model and the sample size of each study, see methods for details. Error bars are standard errors estimated using a binomial model and the sample size of each study, see methods for details.

In Figure 3, we report a collection of cross-correlation plots of the value of $f_{c}$ estimated from the model and the corresponding value of $f_{p}$ measured from autopsies. The rationale behind this plot is that any variations in the metastasis distributions due to geometrical and flow effects should be due to differences in the probabilities that cancer cells reach the target organs. If these geometrical effects are prevalent, we should observe a distinct correlation between $f_{c}$ and $f_{p}$. Inspection of the results reported in Figure 2 shows that most primary tumors display clear correlations between the fraction of simulated cancer cell trajectories reaching the target organs and its effective metastatic colonization as measured by autopsies. In particular, statistically significant correlations are found for lung, colorectal, prostate, pancreatic, and esophago-gastric cancers, whereas no statistically significant correlations can be found for kidney and liver cancer.

**Figure 4:**
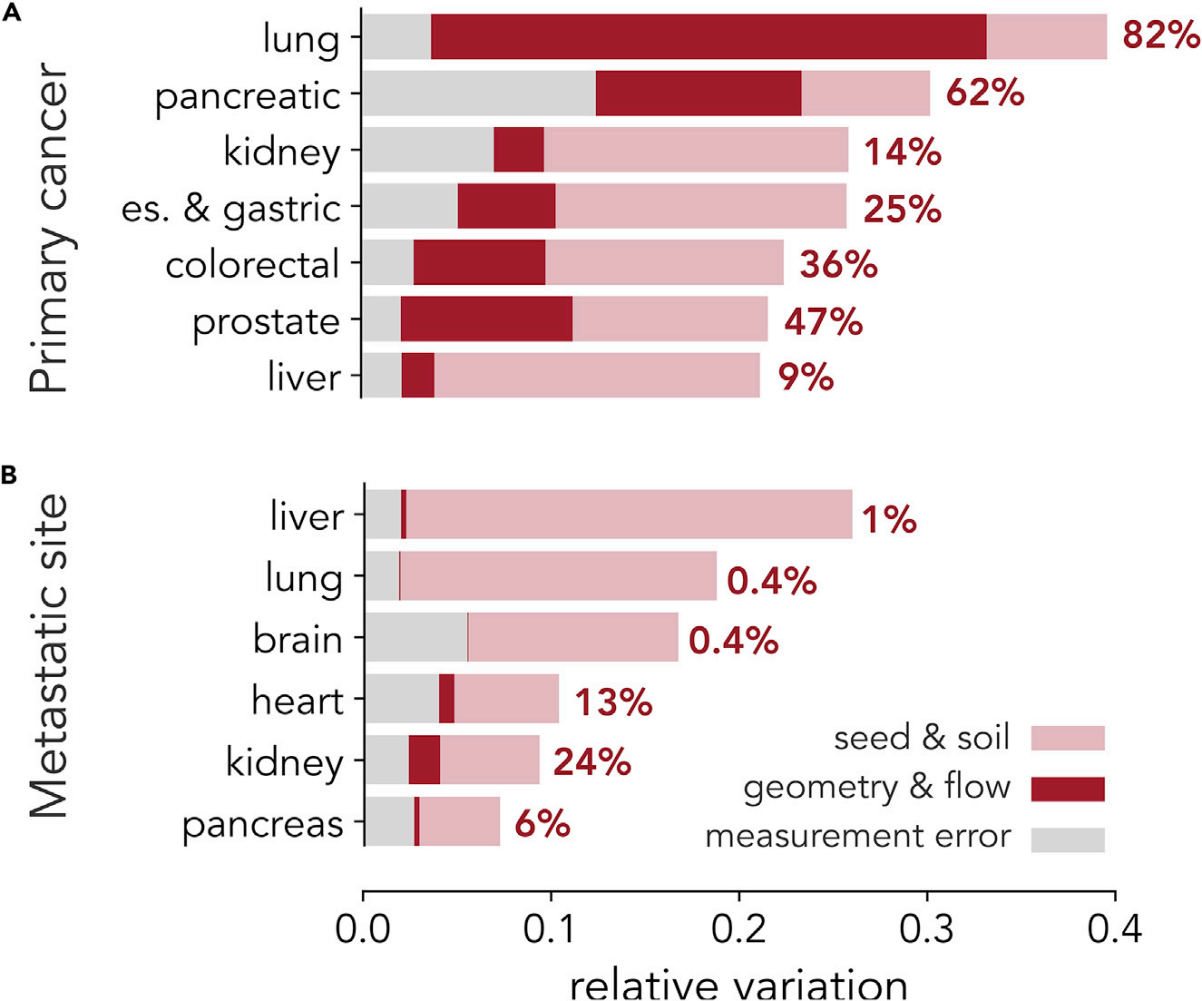
Observed Variations in Metastatic Spread from a Primary Tumor Is Explained Both by “Seed and Soil” and Flow/Geometrical Mechanisms in a Tumor-Dependent Manner (A) Effects due to geometry and flow are found to affect considerably the observed variations in the metastatic sites reached by primary tumors. The extent of the flow contribution depends on the primary tumor and is the largest in lung cancer. (B) The observed variations in primary tumors contributing to metastasis on a given organ is found instead to depend mostly on “seed and soil” mechanisms.

The presence of statistically significant correlations allows us also to estimate how much of the observed variations in the metastatic distribution is explained by our model. The remaining variations are due to seed-soil compatibility and to the measurement errors discussed above. This information is summarized in Figure 4A for different primary tumors. Geometrical factors result to be particularly relevant in the metastatic spread of lung cancer. Although Figure 4A is compiled from the perspective of the primary tumor, we can also take the perspective of the target organ examining the variations in the metastatic patterns with respect to the primary tumors. As shown in Figure 4B, for all the target organs considered, the variations in metastasis can be mostly attributed to seed-soil compatibility, with the possible exception of kidney metastasis, showing a significant dependence on the flow and geometric factors.

## Discussion

In this paper, we have introduced a high-resolution model for the spread of CTCs through the circulatory system and used it to estimate the contribution of blood flow to metastatic spread. Our model expands the scope of earlier simplified models of blood circulation (Scott et al., 2014, Poleszczuk et al., 2016) by a more accurate simulation of the circulatory system. We have restricted our analysis to a set of target organs for which data were available, but our strategy is very general. Detailed statistical data on the precise localization of metastasis are unfortunately not always available. For instance, metastasis to bone and skin are usually recorded without providing information on where they occur.

In conclusion, our computational humanoid model of CTC dynamics allows simulation of the metastatic spread in a realistic geometry, including adhesion mechanisms, and can thus provide guidance for precision medicine to fight metastasis. In this context, experimental recordings of blood flow profiles by contrast-enhanced computed tomography or MRI might be used to identify future sites of metastasis that could be exploited for diagnostics and following them during treatment. Our model could also be expanded along different directions. For example, we could follow the trajectories of other relevant bodies through the circulation systems, such as atherosclerotic plaques or drug carriers.

### Limitations of the Study

In this study we only considered blood circulation and not the lymphatic system. Therefore, we cannot describe the contribution of metastasis spreading through the lymphatic system. This might be important for tumors like melanoma. Further limitations are due to the approximations employed to simulate circulation in the veins where we did not consider valves. At the level of this study, it is not a critical limitation.

## Methods

All methods can be found in the accompanying Transparent Methods supplemental file.

## Data and code Availability

All the simulations are performed with a custom-made Python code. A concise illustration of the algorithm, including a flowchart for the simulation of CTC trajectories, is reported in Figure S4. The simulations code, the circulation network, and all the data extracted from the literature and used to produce results and figures presented in this paper are available at https://github.com/ComplexityBiosystems/CTC-model.

## Supplemental Information

Document S1. Transparent Methods and Figures S1–S4
