# Supplementary material for: Blood Flow Contributions to Cancer Metastasis
Source: iScience. 2020 Apr 18;23(5):101073. doi: 10.1016/j.isci.2020.101073 (PMC7200936; doi:10.1016/j.isci.2020.101073)
Supplement: Document S1. Transparent Methods and Figures S1–S4 [file mmc1.pdf]

iScience, Volume *23*

Supplemental Information

Blood Flow Contributions

to Cancer Metastasis

Francesc Font-Clos, Stefano Zapperi, and Caterina A.M. La Porta

Supplemental Information: Blood flow contributions to cancer metastasis

Francesc Font-Clos[a], Stefano Zapperi[a,b], Caterina A. M. La Porta[c,d,*]

[a]*Center for Complexity and Biosystems, Department of Physics, University of Milan, Via Celoria 16, 20133 Milano, Italy*
[b]*CNR - Consiglio Nazionale delle Ricerche, Istituto di Chimica della Materia Condensata e di Tecnologie per l'Energia, Via R. Cozzi 53, 20125 Milano, Italy*
[c]*Center for Complexity and Biosystems, Department of Environmental Science and Policy, University of Milan, via Celoria 26, 20133 Milano, Italy*
[d]*CNR - Consiglio Nazionale delle Ricerche, Istituto di Biofisica, via Celoria 26, 20133 Milano, Italy*

Transparent Methods

Anatomical data

To build an accurate network representation of arterial and venous circulatory systems, we use data from the Anatomography project, using its underlying dataset, BodyParts3D (Mitsuhashi et al., 2009), a set of 2234 mesh files covering a full 3D whole-body model obtained from 2mm interval magnetic resonance imaging (MRI) images of a male subject. Each mesh file is associated with terms from the Foundation Model of Anatomy (FMA), a domain ontology of anatomical knowledge. Using FMA terms, we are able to locate 639 mesh files corresponding to the arterial circulatory system (keywords: artery, arteries, arterial, aorta) and 395 mesh files corresponding to the venous circulatory system (keywords: vein, veins, venous, cava). We subdivide each mesh into its connected components, obtaining 1319 meshes, and remove 53 duplicated meshes. In summary, we retrieve a total of 1266 mesh files from the BodyParts3D dataset that cover the full circulatory system. We also locate 16 meshes that correspond to the following body organs: brain, large intestine, small intestine, left lung, right lung, pancreas, stomach, heart, left kidney, right kidney, liver, prostate, gallbladder, urinary bladder, left adrenal gland and right adrenal gland.

3D mesh to graph conversion

In order to transform a mesh into a graph (see Fig. 1a), we use Elastic Principal Graph (ElPiGraph) (Albergante et al., 2018), a manifold learning algorithm originaly designed to infer branching trajectories in single-cell datasets. Specificaly, we sample 5000 points uniformly across the surface of the mesh and feed those to ElPiGraph. We manually verify that the obtained graph corresponds to the correct topological structure of the mesh. In cases of meshes with complicated geometry, however, ElPiGraph fails to

*Corresponding author: caterina.laporta@unimi.it

obtain the correct graph. To tackle those cases, we split the mesh into two pieces using the sign of its first principal component, obtaining two submeshes with simpler geometry. We iterate this process until all submeshes can be correctly transformed into a graph using ElPiGraph, and reconstruct the graph of the original mesh by joining the obtained graphs along the planes that where used to split the mesh. In addition, we measure the radius of the vessel at each edge by fitting the smallest possible circumference centered at the middle point of the edge. In summary, for each 3D-mesh we obtain a graph whose nodes have an associated position and whose edges have an associated radius.

Construction of the full-body graph

We merge the 1266 graphs obtained from BodyParts3D mesh files to obtain a single full-body graph composed of 23285 nodes and 23804 edges. The details of the graph-merging process are as follows: first, we automatically join graphs whose associated meshes have non-null intersection throught their closest nodes. Then, we manually verify them by visualizing their associated meshes, making sure they correspond to anatomically connected vessels and removing the connection when that is not the case. While for small vessels in some cases it is difficult to assert if two graphs should be connected or not, we take special care when dealing with major arteries and veins, and have verified that the main known circulatory paths are correctly assembled in the final whole-body graph. Finally, we add nodes representing the body organs for which a mesh file is available, and connect them to nodes that verify one of the two following conditions: (1) they are inside the mesh of the organ, or (2) they are at a distance of less than 10mm from the organ. The distance between an organ and a node is computed as the shortest distance between the node and any vertex of the mesh of the organ. The BodyParts3D mesh files are incomplete, particularly for the head section which lacks all the venous system and parts of the common carotid arteries, connecting the head to the neck. We thus reconnect the left and right common carotid arteries to the corresponding internal and external carotid arteries. It is less straightforward to replace the missing veins, but we can not disregard completely the veins in the head because this would lead to incorrect global flow patterns. We solve this problem by adding a set of effective head vessels, tuning their flow resistance so that systemic circulation is accurately reproduced.

Solution to hemodynamic flow equations

We assume that blood is a Newtonian fluid of constant viscosity and that vessels are essentially non-deformable, which is a good approximation for large and medium size vessels (Quarteroni, 2006). Pressure changes due to the systole and diastole periods during a full cardiac cycles are not included in the model. We assume a constant mean arterial pressure of 100 mmHg for the main ciruclatory system and 14 mmHg for the pulmonary circulatory system. This approximation is justified since the cardiac cycle occurs in a timescale of the order of seconds, while we are interested in metastatic patterns of CTCs, happening in the course of much larger timescales. Furthermore, given that the dynamics of our simulated cancer cell trajectories depend on ratios of blood flow rates, and not on their absolute values, the fraction of cells measured in simulations is effectively independent of the pressure and viscosity values. Finally, to account for the effect of the vessels not included in the original mesh data, we add resistive elements at the arterial-to-venous connections. We use the Powell method as implemented in the

scientific python library scipy (Jones et al., 2001) and data from (Williams and Leggett, 1989) to adjust the effective resistance of these connections, guaranteeing a realistic blood flow distribution, as shown in Fig. 1.

Under those assumptions, we consider a system of hemodynamic flow equations on a network of N nodes, divided into B leaf nodes and $N - B$ internal nodes. Our setup considers (i) flow conservation for all internal nodes and (ii) the Hagen-Poiseuille equation for all edges. For a given edge (i, j), the Hagen-Poiseuille equation reads:

$$J_{ij} = \frac{\pi \Delta p_{ij} R_{ij}^4}{8 \eta L_{ij}} \tag{1}$$

where J_{ij} stands for the flow rate along edge (i, j), R_{ij} is the measured radius of the edge, L_{ij} its length and η the viscosity of the fluid, which we assume constant. The pressure difference Δp_{ij} is simply

$$\Delta p_{ij} = p_j - p_i \tag{2}$$

with p_i, p_j the pressure at nodes i and j. We impose blood flow conservation for all internal nodes i,

$$\sum_j J_{ij} = 0, \qquad i = 1 \ldots N - B \tag{3}$$

and set boundary conditions for the pressure of the remaining B leaf nodes, which correspond to connections to/from the heart. Combining these three equations we obtain a linear system of equations,

$$\sum_j \frac{\pi (p_j - p_i) R_{ij}^4}{8 \eta L_{ij}} = 0, \qquad i = 1 \ldots N, \tag{4}$$

Given that the values of R_{ij} and L_{ij} are known, and that the pressure of B nodes is fixed by boundary conditions, we are left with $N - B$ variables and $N - B$ linear constraints. The topology of the network ensures that the system has a solution, which we find using the sparse.linalg.sparse_inv function from the scientific python library scipy (Jones et al., 2001). Finally, we calculate the flux along the edges J_{ij} by inserting the obtained pressure values p_i into Eq. (1). We solve the system separately for the main circulatory system and the pulmonary circulatory system to allow for realistic different pressure differences on each system when setting boundary conditions. In simulating venous circulation, we do not consider the presence of valves.

Simulation of cancer cell trajectories

We simulate cancer cell trajectories assuming that cancer cells follow the blood flow. To simulate metastasis stemming from a given primary tumor, cells are initially released from random nodes belonging to the relevant body organ. At branching points, cells choose which branch to take with probabilities proportional to the blood flow of each branch. That is, given a node i with $j = 1 \ldots k_i$ outgoing edges, the probability for a cancer cell to choose edge j is given by

$$P_{i \to j} = \frac{J_{ij}}{\sum_j J_{ij}} \tag{5}$$

where J_{ij} is the blood flux along edge (i, j). Cell trajectories start from nodes that are closer than 10 mm from a given organ and follow the blood flow as described. We also assume that cells can enter into capillary beds with rate ϵ only if found at a distance less than δ from the wall, while they can not enter otherwise (see Fig. 1). When cells reach the outlet of an artery and do not attach to the capillary bed, they are re-inserted in the nearest node in venous system. For a short vessel segment, blood flow can be approximated by the equations of laminar flow in a tube (Glaser, 1999). In this case, the velocity profile $v(r)$ in a tube of radius R and length L is known to be of parabolic type,

$$v(r) = \frac{\Delta p}{4L\nu}(R^2 - r^2), \tag{6}$$

We use this expression to compute the probability P_{wall} that a cell flowing along the blood stream is at a distance less than δ from the wall of a vessel of radius R:

$$P_{\text{wall}} = \frac{\int_0^{2\pi} d\theta \int_{R-\delta}^{R} dr\, r v(r)}{\int_0^{2\pi} d\theta \int_0^{R} dr\, r v(r)} \simeq \frac{4\delta^2}{R^2} \tag{7}$$

Multiplying then by the rate ϵ, we obtain an expression for the rate for a cell to enter into a capillary bed from a vessel of radius R:

$$P_{\text{exit}}(R) = P_{\text{wall}} \cdot \epsilon = \frac{4\delta^2 \epsilon}{R^2} \tag{8}$$

This expression accounts for the possibility that cancer cells enters into a capillary bed and reflects the fact that when going through large vessels, cells are less likely to be near the walls and thus less likely to enter into capillaries. The value of ϵ would in principle depend on the properties of tumor cells, while the value of δ could be related to the size of circulating tumor cells and the clusters they form. We do not model these aspects in this work, since our aim is to disentangle seed-soil versus geometry and flow contributions to cancer metastasis incidence rates. We thus choose both δ and ϵ to be constant across the vascular system.

Cell attachment in capillary beds

Once a CTC enters into a capillary bed, we can estimate the probability of extravasation to seed a new metastasis. To this end, we model capillary beds as binary trees obeying on Murray's law (Welter and Rieger, 2010) which states that if a vessel of radius R splits in two vessels of radii R_a and R_b, then $R^3 = R_a^3 + R_b^3$ (Murray, 1926). If the inlet to the capillary bed has radius R_0 and $R_a = R_b$, after n bifurcations the capillary radius R_n is given by (Sherman, 1981)

$$R_n = 2^{-1/3} R_{n-1} = \cdots = 2^{-n/3} R_0, \tag{9}$$

Furthermore, we assume that the lengths of the vessel are proportional to their radius $L_n = \alpha R_n$, as suggested by morphometric data Huang et al. (1996). To obtain the total number of bifurcations N in a capillary bed, we set $R_N = 3.5\mu$m, based on the observation that the smallest capillaries have a diameter of $5 - 10\mu$m Mittal et al. (2005); Chan et al. (2012). Then using Eq. 9, we obtain

$$N = 3 \log_2 \left(\frac{R_0}{R_N} \right) \tag{10}$$

We next assume that the attachment probability while traversing a capillary in the tree is proportional to Eq. 8 multiplied by the time spent in the capillary. Since the flow after each bifurcation is conserved (i.e. $Q_n = 2Q_n$) in a *Murray's tree*, the time T_n to traverse a capillary at generation n is independent of n. Therefore, the probability of attaching while traversing the n-th generation of the tree is simply given by:

$$P(n) = C/R_n^2 \tag{11}$$

The total probability of attachment while traversing the whole tree can be approximated as follows:

$$P_{\text{ATT}} = 1 - \prod_{n=1}^{N} (1 - P(n)) \simeq 1 - \exp\left(-\sum_{n=1}^{N} P(n)\right) \tag{12}$$

where the approximation is valid as long as $P(n)$ is small. Now the sum can be evaluated explicitly,

$$\sum_{n=1}^{N} P(n) = \sum_{n=1}^{N} \left(\frac{C}{R_0^2}\right) 2^{2n/3} \tag{13}$$

$$= \frac{C}{R_0^2} \sum_{n=1}^{N} \left(2^{2/3}\right)^n \tag{14}$$

$$= \frac{C}{R_0^2} \frac{2^{2/3}\left(2^{2N/3} - 1\right)}{2^{2/3} - 1} \tag{15}$$

substituting eq. (10) back and condensing all the prefactors into a single constant C_0, we obtain

$$\sum_{n=1}^{N} P(n) = \frac{C_0}{R_0^2}\left(\frac{R_0^2}{R_N^2} - 1\right) = C_0\left(\frac{1}{R_N^2} - \frac{1}{R_0^2}\right) \tag{16}$$

so that the attachment probability for a tree of inlet radius R_0 is approximately given by

$$P_{\text{ATT}} \simeq 1 - \exp\left(-C_0\left(\frac{1}{R_N^2} - \frac{1}{R_0^2}\right)\right). \tag{17}$$

In the simulations, we chose the inlet radii to be $R_0 = 100\mu$m, except for vessels whose radius is already smaller that R_0 where we use the vessel radius itself. Finally, we set $C_0 = 10^{-2}$mm^2.

Morphometric analysis

We define the number of generations from any vessel segment to the heart as the number of branching points along the shortest path. The shortest path is computed using the metric distance along it via the networkx python library. For capillaries, the total number of generations is computed as the number of generations to the heart plus the number of generations given in Eq. 10. Fig S1(d) shows the distribution of total number of generations, whose median value 31.7 is in agreement with known morphometric measurements (Huang et al., 1996; Mittal et al., 2005).

Datasets

We collect data on frequency of metastasis among different primary tumors and corresponding metastatic sites from 5 large autopsy studies: **1. (Abrams, 1950)** Study of 1000 epithelial malignant neoplasm cases, autopsied at Montefiore Hospital, New York, between 1943 and 1947 (Abrams et al., 1950). We collect the tabulated data of frequency of metastasis among 39 metastatic sites for 167 breast cancer cases, 118 colon cancer cases, 34 kidney cancer cases, 64 ovary cancer cases, 32 pancreatic cancer cases, 87 rectuom cancer cases and 119 gastric cancer cases. The colorectal data displayed in Fig. S3 are obtained pulling together colon and rectum cases . **2. (Bubendorf, 2000)** Study of 1589 prostate cancer cases, autopsied at the Institute of Pathology of the University of Basel between 1967 and 1995, of which 556 reveal hematogeneous metastasis over 17 different metastatic sites (Bubendorf et al., 2000). **3. (diSibio, 2008)** Review study of data from 3827 autopsies performed between 1914 and 1943 at 5 different medical centers in the state of Massachusetts (Disibio and French, 2008). The study includes data for 41 primary tumors and 30 different metastatic sites. We collect tabulated metastasis frequency data for 437 rectum cancer cases, 432 breast cancer cases, 418 cervix cancer cases, 348 gastric cancer cases, 193 prostate cancer cases, 183 bladder cancer cases, 165 tongue cancer cases, 163 lung cancer cases, 129 esophageal cancer cases, 123 colon cancer cases, 120 uterus cancer cases, 117 pharynx cancer cases, 109 pancreatic cancer cases and 28 other primary tumors with less than 100 cases. The colorectal and gastric and esophageal data displayed in Fig S3 are obtained pulling together colon and rectum cases and gastric and esophaegeal cases, respectively. **4. (Budczies, 2015)** Study of 1008 cancer cases with metastatic solid malignancies autopsied at the Charité Institute of Pathology, Berlin, between 2000 and 2013 (Budczies et al., 2015). The study comprises 16 different primary tumors and records 20 different metastatic sites. We collect values for relative frequency of metastasis from the main text in some cases, and infer the rest from their Figure 3A, for 280 lung cancer cases, 98 esophageal and gastric cancer cases, 89 breast cancer cases, 89 colorectal cancer cases, 78 pancreatic cancer cases, 71 biliary cancer cases, 48 head and neck cancer cases, 46 kidney cancer cases, 40 neuroendocrine cancer cases, 33 prostate cancer cases, 32 liver cancer cases and 4 other primary tumors with less than 30 cases. **5. (Schlageter, 2016)** Study of 398 hepatocellular carcinoma cases autopsied at the Institute of Pathology, Basel, between 1969 and 1983 and between 1988 and 2012 (Schlageter et al., 2016). The study records 11 different metastatic sites.

Computation of corrected r^2 values

The regression lines and corresponding r^2 values in Fig. 3 are computed in logarithmic space and taking into account measurement errors in the metastatic frequency data. We limit ourselves to combinations of primary tumor and metastatic site for which at least two independent studies are available. To compute regression in logarithmic space, we consider the standard linear regression equation, $\hat{y} = \alpha x + \beta$ with $x = \log(f_c)$ the logarithm of the fraction of cells obtained from the circulation model, $y = \log(f_p)$ the logarithm of the fraction of patients obtained from the literature and $\hat{y} = \alpha \log(f_c) + \beta$ the linear regression estimation. After fitting the slope α and intercept β using the stats.linregress function from from the scientific python library scipy (Jones et al., 2001), we compute the corrected explained variance ratio r^2,

$$r^2 = \frac{S^2 - E^2}{S^2 - e^2}. \tag{18}$$

Notice how this calculation differs from the more usual R^2 one in that the denominator of Eq. (18) has an additional $-e^2$ term that accounts for measurement errors. In this expression E^2, S^2 and e^2 are defined as follows:

$$E^2 = \frac{1}{N}\sum_i (\hat{y}_i - y_i)^2 \tag{19}$$

$$S^2 = \frac{1}{N}\sum_i (y_i - \bar{y})^2 \tag{20}$$

$$e^2 = \frac{1}{N}\sum_i \sigma_{y_i}^2 \tag{21}$$

The logarithmic measurement errors σ_y are computed from the linear measurement errors σ_{f_p} via the error propagation formula,

$$\sigma_y = \left|\frac{\sigma_{f_p}}{f_p}\right|. \tag{22}$$

Linear measurement errors are inferred from the number of patients N_p of each dataset assuming a binomial model,

$$\sigma_{f_p} = \sqrt{\frac{f_p(1-f_p)}{N_p}}. \tag{23}$$

Decomposition of variability of metastasis incidence rate

The values of S^2, E^2 and e^2 are also used in Fig. 4 to decompose the total variability of metastasis incidence rates S^2 into three pieces:

$$S^2 = \underbrace{(S^2 - E^2)}_{\text{geom \& flow}} + \underbrace{(E^2 - e^2)}_{\text{seed \& soil}} + \underbrace{(e^2)}_{\text{measurement}} \tag{24}$$

Given that our model is based on geometry and flow and does not incorporate any specific target-organ compatibility factors, these three terms can be interpreted in terms of seed & soil and geometry & flow hypothesis: $S^2 - E^2$ is the variability of the data that the model can account for, and hence is attributed to the geometry and flow hypothesis; $E^2 - e^2$ is the variability of the data that the model cannot account for after taking into account measurement errors, and is attributed to the seed and soil hypothesis; and e^2 is by definition the measurement error of the data.

Data and code availability

All code and data necessary to reproduce the results and figures of this paper are available at `https://github.com/ComplexityBiosystems/CTC-model`. This includes the circulatory network, python code to simulate CTC trajectories, and the autopsy data extracted from the literature.

Supplemental references

Abrams, H. L., Spiro, R., Goldstein, N., Jan 1950. Metastases in carcinoma; analysis of 1000 autopsied cases. Cancer 3 (1), 74–85.

Albergante, L., Mirkes, E. M., Chen, H., Martin, A., Faure, L., Barillot, E., Pinello, L., Gorban, A. N., Zinovyev, A., Apr. 2018. Robust and scalable learning of complex dataset topologies via elpigraph.

Bubendorf, L., Schöpfer, A., Wagner, U., Sauter, G., Moch, H., Willi, N., Gasser, T. C., Mihatsch, M. J., May 2000. Metastatic patterns of prostate cancer: an autopsy study of 1,589 patients. Hum Pathol 31 (5), 578–83.

Budczies, J., von Winterfeld, M., Klauschen, F., Bockmayr, M., Lennerz, J. K., Denkert, C., Wolf, T., Warth, A., Dietel, M., Anagnostopoulos, I., Weichert, W., Wittschieber, D., Stenzinger, A., Jan 2015. The landscape of metastatic progression patterns across major human cancers. Oncotarget 6 (1), 570–83.

Chan, G., Balaratnasingam, C., Paula, K. Y., Morgan, W. H., McAllister, I. L., Cringle, S. J., Yu, D.-Y., 2012. Quantitative morphometry of perifoveal capillary networks in the human retina. Investigative ophthalmology & visual science 53 (9), 5502–5514.

Disibio, G., French, S. W., Jun 2008. Metastatic patterns of cancers: results from a large autopsy study. Arch Pathol Lab Med 132 (6), 931–9.

Glaser, R., 1999. Biophysics, 1st Edition. Springer-Verlag Berlin Heidelberg.

Huang, W., Yen, R. T., McLaurine, M., Bledsoe, G., Nov 1996. Morphometry of the human pulmonary vasculature. J Appl Physiol (1985) 81 (5), 2123–33.

Jones, E., Oliphant, T., Peterson, P., et al., 2001. SciPy: Open source scientific tools for Python. Http://www.scipy.org/.
URL `http://www.scipy.org/`

Mitsuhashi, N., Fujieda, K., Tamura, T., Kawamoto, S., Takagi, T., Okubo, K., Jan. 2009. BodyParts3D: 3D structure database for anatomical concepts. Nucleic Acids Res. 37 (Database issue), D782–5.

Mittal, N., Zhou, Y., Ung, S., Linares, C., Molloi, S., Kassab, G. S., Aug 2005. A computer reconstruction of the entire coronary arterial tree based on detailed morphometric data. Ann Biomed Eng 33 (8), 1015–26.

Murray, C. D., 1926. The physiological principle of minimum work: I. the vascular system and the cost of blood volume. Proceedings of the National Academy of Sciences of the United States of America 12 (3), 207.

Quarteroni, A., 2006. What mathematics can do for the simulation of blood circulation. MOX Report.

Schlageter, M., Quagliata, L., Matter, M., Perrina, V., Tornillo, L., Terracciano, L., 2016. Clinicopathological features and metastatic pattern of hepatocellular carcinoma: An autopsy study of 398 patients. Pathobiology 83 (6), 301–7.

Sherman, T. F., 1981. On connecting large vessels to small. the meaning of murray's law. The Journal of general physiology 78 (4), 431–453.

Welter, M., Rieger, H., 2010. Physical determinants of vascular network remodeling during tumor growth. The European Physical Journal E 33 (2), 149–163.

Williams, L. R., Leggett, R. W., Aug. 1989. Reference values for resting blood flow to organs of man. Clin. Phys. Physiol. Meas. 10 (3), 187–217.

Supplemental figures

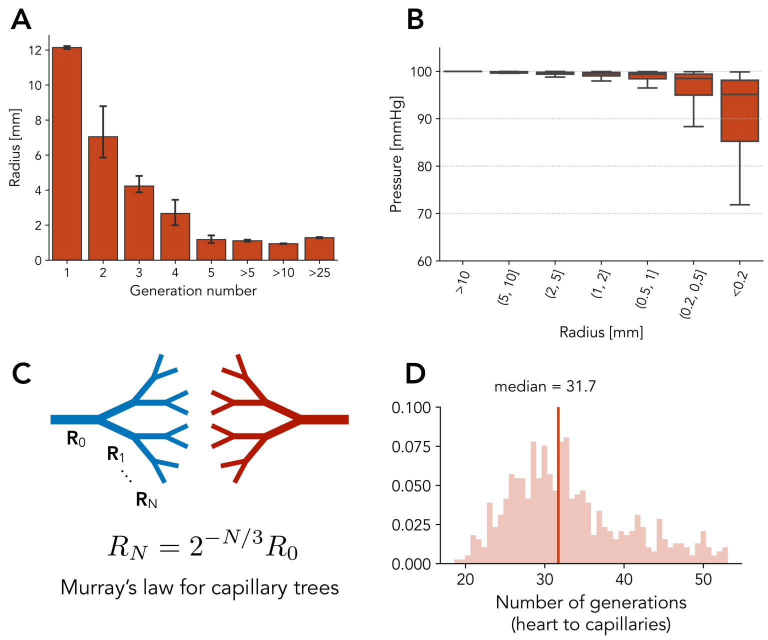

Figure S1: **Morphological details fo the MRI-derived circulatory network model. Related to Fig. 1.** (a) Measured radii as a function of generation number, defined as the number of branching points from a vessel segment to the heart. The panel shows that vessels further away from the heart tend to be thinner, as expected. (b) Mean arterial pressure (MAP) obtained solving the blood flow equations, as a function of the radius of arterial vessels. The pressure at the heart is fixed to 100 mmHg. The panel shows that in the range 10 to 1 mm, pressure is maintained close to 100 mmHg, displaying only a mild drop with decreasing radius as expected. For very small vessels of radii below 0.5 mm, we observe instead is a larger pressure drop. (c) Schematic representation of our modeling of capillary beds using Murrays law. (d) Distribution of the number of generations from heart to capillaries. Generations are defined as number of branching points along a shortest path.

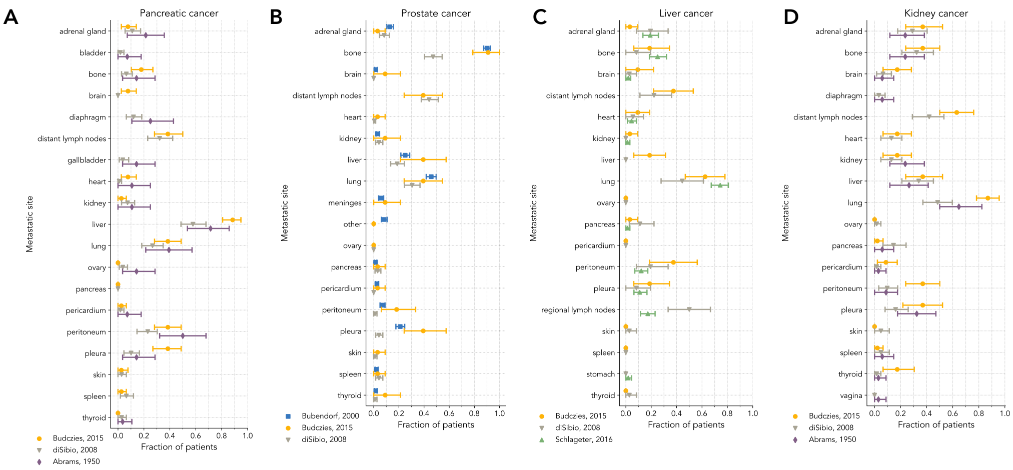

Figure S2: **Statistics of data from autopsies (First part). Related to Fig. 3.** A compilation of the values of f_p — the fraction of patients with a given primary tumor and metastasis found in a distant organ – obtained from autopsy data reported in the literature for a) pancreatic cancer, b) prostate cancer, c) liver cancer and d) kidney cancer. Error bars are standard errors estimated using a binomial model and the sample size of each study, see methods for details.

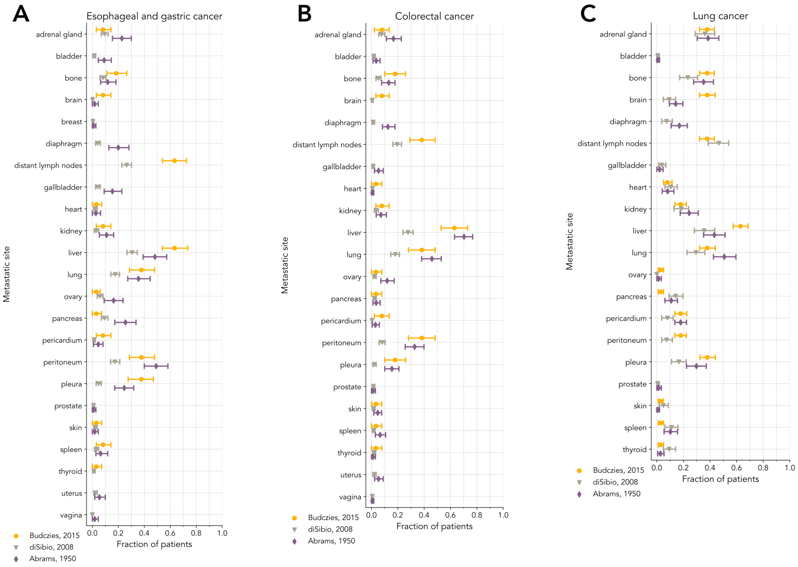

Figure S3: **Statistics of data from autopsies (Second part). Related to Fig. 3.** A compilation of the values of f_p — the fraction of patients with a given primary tumor and metastasis found in a distant organ – obtained from autopsy data reported in the literature for a) esophageal and gastric cancer, b) colorectal cancer and c) lung cancer. Error bars are standard errors estimated using a binomial model and the sample size of each study, see methods for details.

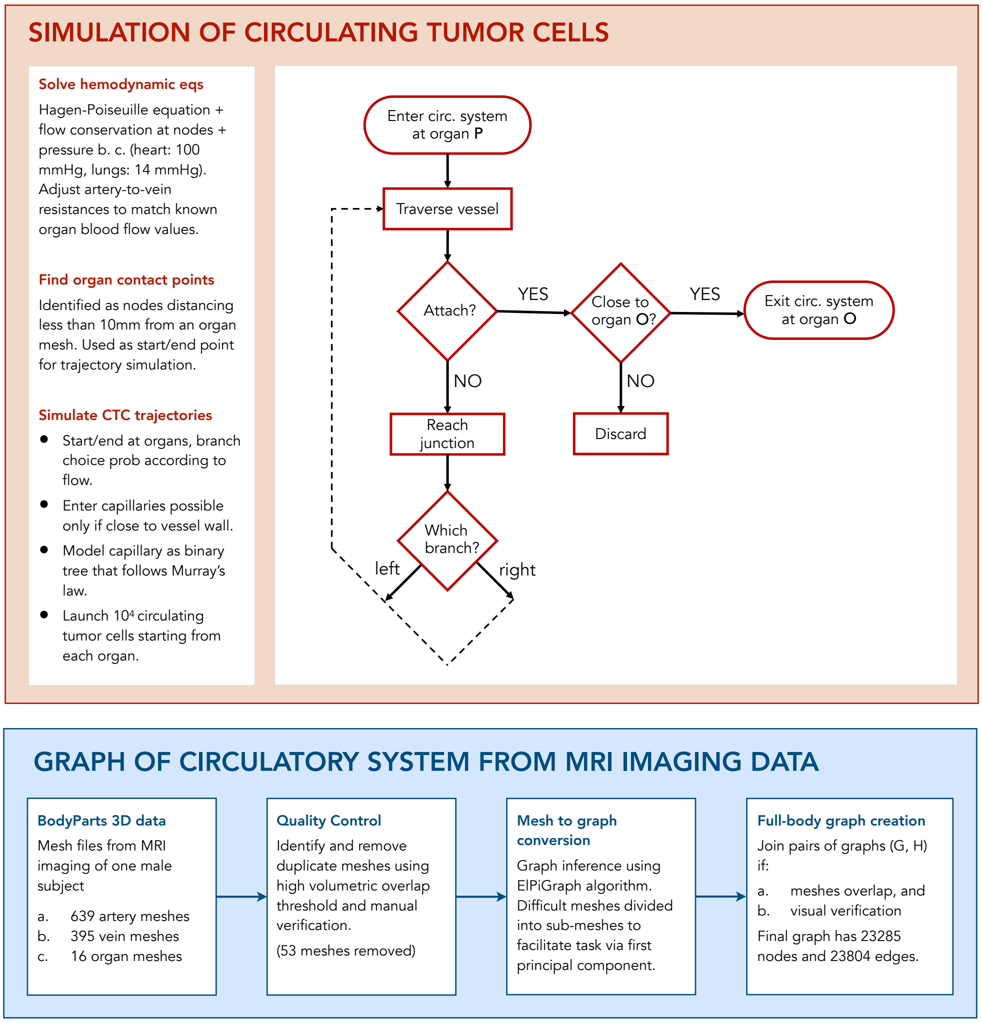

Figure S4: **A schematic description of the algorithms used for the simulations. Related to Fig. 2.**
